# A simple genotyping method to detect small CRISPR-Cas9 induced indels by agarose gel electrophoresis

**DOI:** 10.1038/s41598-019-39950-4

**Published:** 2019-03-14

**Authors:** Debanjan Bhattacharya, Erwin G. Van Meir

**Affiliations:** 1 Laboratory of Molecular Neuro-Oncology, Departments of Neurosurgery and Hematology & Medical Oncology, Emory University School of Medicine, Atlanta, GA 30322 USA; 2 Winship Cancer Institute, Emory University, Atlanta, GA 30322 USA

## Abstract

CRISPR gene editing creates indels in targeted genes that are detected by genotyping. Separating PCR products generated from wild-type versus mutant alleles with small indels based on size is beyond the resolution capacity of regular agarose gel electrophoresis. To overcome this limitation, we developed a simple genotyping method that exploits the differential electrophoretic mobility of homoduplex versus heteroduplex DNA hybrids in high concentration agarose gels. First, the CRISPR target region is PCR amplified and homo- and hetero-duplexed amplicons formed during the last annealing cycle are separated by 4–6% agarose gel electrophoresis. WT/mutant heteroduplexes migrate more slowly and are distinguished from WT or mutant homoduplexes. Heterozygous alleles are immediately identified as they produce two distinct bands, while homozygous wild-type or mutant alleles yield a single band. To discriminate the latter, equal amounts of PCR products of homozygous samples are mixed with wild-type control samples, subjected to one denaturation/renaturation cycle and products are electrophoresed again. Samples from homozygous mutant alleles now produce two bands, while those from wild-type alleles yield single bands. This method is simple, fast and inexpensive and can identify indels >2 bp. in size in founder pups and genotype offspring in established transgenic mice colonies.

## Introduction

CRISPR-Cas9 based gene editing has become the method of choice for rapid generation of cell lines and transgenic animals with targeted genomic alterations, thereby greatly facilitating genetic engineering for understanding gene and protein function. CRISPR (clustered regularly interspaced short palindromic repeats) gene editing uses the endonuclease Cas9 combined with a guide RNA (gRNA) designed to cleave a targeted genomic sequence and induce a double-strand DNA break^1,2^. Cells or single cell zygotes commonly repair Cas9 cleaved DNA using the error-prone non-homologous end joining (NHEJ) repair pathway, which randomly inserts or deletes DNA bases to repair double-strand breaks^3^. The most common indels from NHEJ repair range from 5–9 nucleotides^3^.

To detect cell line clones or transgenic animals that carry CRISPR engineered deletions, simple yet efficient strategies for genotyping are needed. So far, the gold standard for detecting modified genes has been Sanger sequencing of PCR products of the targeted gene region, either directly as mixed alleles or after their cloning as single alleles. Other methods include the T7 endonuclease-I (T7E1) cleavage assay^4^, PAGE based assays to detect heteroduplexes^5^ and qPCR based assays^6^. However, all these methods are either expensive or require substantial turnaround time and sophisticated instruments. Here we present an alternative simple two-step heteroduplex analysis method involving PCR of genomic DNA and agarose gel electrophoresis to genotype tissue or cells with CRISPR induced small allelic indels. Hybridization of two strands of DNA that are not perfectly complementary leads to bending and small single strand protrusions in the mismatched region, which increase the heteroduplex surface area and restricts its motility through a sieving matrix^7–9^. Heteroduplex analysis has been previously used in SNP and indel genotyping in mice using PAGE^5,10,11^. We now demonstrate that homoduplex and heteroduplex DNA fragments carrying small indels also display differences in migration when subjected to 4–6% agarose gel electrophoresis.

To demonstrate our method, we have implemented it for three applications: (i) identify genetically altered founder pups from CRISPR-Cas9 mediated targeting of exon 10 of the mouse *Adgrb3* gene, (ii) genotype the offspring of heterozygous *Adgrb3*+/− mice carrying a CRISPR-Cas9 induced 7-nucleotide deletion, which results in generation of a stop codon (unpublished data), and (iii) genotype the offspring of mice carrying a CRISPR-Cas9 induced 3-nucleotide insertion in exon 15 of the mouse *Scn8a* gene. The *Adgrb3* gene belongs to the adhesion GPCR subfamily and has a wide array of roles in biology and disease^12^. The *Scn8a* gene encodes a protein that forms the ion pore region of the alpha subunit of voltage gated sodium channel Na_v_1.6, which is widely expressed in neurons^13^.

## Methods

### Genomic DNA isolation

Mice tail snips (4 mm) were collected in sterile 1.5 ml microcentrifuge tubes (MSP 78-100339). 100 µl of a tissue digestion buffer (NaOH 25 mM and EDTA 0.2 mM) was added to each sample and heated for 1 hour at 100 °C in a heating block (Isotemp, Fisher Scientific). Following digestion, 100 µl of neutralization buffer (40 mM Tris buffer, pH 7.5) was added. The genomic DNA concentration in resulting solution (200 µl) was measured using a spectrophotometer (Nanodrop 200 C, Thermo Scientific). Expected DNA concentration was 60–80 ng/µl depending on size of tail tissue. DNA concentration was adjusted to 50 ng/µl in all samples by adding nuclease-free water. Isolated DNA samples can be stored in microcentrifuge tubes at −20 °C before subsequent steps.

### PCR amplification of the CRISPR targeted region

**Figure 1:**
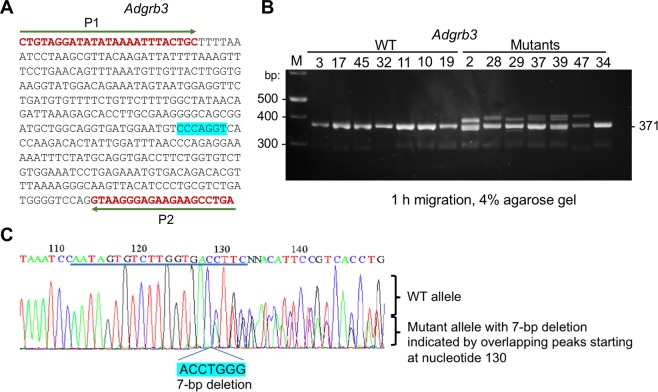
Genotyping of transgenic mice obtained through CRISPR-Cas9 targeting of the *Adgrb3* gene. (**A**) Sequence of the PCR amplified region encompassing the CRISPR target in exon 10 of mouse *Adgrb3* gene. Amplicons of 371 bp. (WT) and 364 bp. (mutant) are obtained. The 7 nucleotides deleted by CRISPR-Cas9 are indicated in blue. The positions of the two primers (P1 and P2) used for PCR amplification are shown (see primer sequences in Table 1). (**B**) Screening of F0 pups generated from CRISPR-Cas9 microinjected zygotes implanted in pseudo-pregnant females to detect potential heterozygous founder animals. Results of 14 of 47 pups are shown. The CRISPR targeted *Adgrb3* locus was PCR amplified from pup genomic DNA using primers P1/P2 and PCR products were size-separated by electrophoresis on a 4% agarose gel for 1 h. The expected amplicon size is 371-bp for WT samples. Sequencing of samples confirmed WT or mutant status (Supplementary Table 1). All mutants yielded heteroduplex bands, except for #34, which has a 1-bp insertion. (**C**) Sequencing of PCR amplicon of founder pup (#16) evidenced a 7-bp. deletion in the CRISPR target region (underlined).

Design a pair of primers (~20 bp in size) that amplifies a 150–400 bp region spanning the gene deletion/insertion area using NCBI primer design tool (https://www.ncbi.nlm.nih.gov/tools/primer-blast/). Smaller amplicons (<200 bp) are preferable for smaller indels (~3 nucleotides). Sequences of primers used are presented in Table 1 and target regions for *Adgrb3* and *Scn8a* genes are shown in Figs 1A and 3A respectively.

**Table 1:** List of sequence of different primer sets (with amplicon sizes) used to amplify CRISPR target regions of *Adgrb3* and *Scn8a* genes.

Gene Targeted	Direction and Name	Primer Sequences (5′ to 3′)	Amplicon size
*Adgrb3*	Forward (P1)	CTGTAGGATATATAAAATTTACTGC	371 bp
	Reverse (P2)	TCAGGCTTCTTCTCCCTTAC	
	Forward (P3)	GTAATGGAGGTTCTGATGTGTTTC	196 bp
	Reverse (P4)	TGTCTGTCACATTTCTCAGGA	
	Forward (P5)	TTCCACAGACACCAGAAGGTCA	100 bp
	Reverse (P6)	AGGGGCAGCGGATGCTGGCAG	
*Scn8a*	Forward (P7)	TTTCAGCAATGTGGGGTAGCA	135 bp
	Reverse (P8)	GCCAACGGAGTTCCCAATGA	
	Forward (P9)	GCAATGTGGGGTAGCAGATT	186 bp
	Reverse (P10)	ACCACGGCAAAGATGAAGAC	

For PCR, 10 µl of GoTaq green master-mix (Promega, M7123) and 1 µL of each of the forward and reverse primers (100 µM stock; Integrated DNA Technologies, USA) pre-diluted to 10 µM were added per PCR tube (0.2 mL; Thermo Scientific). Finally, 5 µl of genomic DNA sample and 3 µl of nuclease-free water were added to each tube for a final reaction volume of 20 µl. PCR was performed using a standard thermocycler (Biometra T3 thermocycler, Jena, Germany). The PCR conditions were as follows: Initial denaturation (95 °C for 3 minutes), followed by 34 cycles of denaturation (94 °C for 30 secs), annealing (59 °C for 30 secs) and extension (72 °C for 30 secs) and a final extension at 72 °C for 4 minutes. PCR products were removed from the thermocycler and maintained at room temperature for a few minutes before loading on the agarose gel.

### Preparation of agarose gels and electrophoresis of PCR products

4% and 6% agarose gels were prepared using low EEO agarose of 95% purity (Sigma-Aldrich, A5093) dissolved in 1X Tris-Acetate EDTA (TAE) buffer (40 mM Tris-acetate, 1mM EDTA, pH 8.3, Fisher Bioregents BP13324) by heating the solution in a microwave oven for 2–3 minutes. 5 µl of ethidium bromide (10 mg/ml) (OmniPur, Calbiochem, 4410) was added to the melted agarose, and it was immediately poured on a UV transparent gel casting tray of 15 × 10 cm size (BioRad, 1704416) fitted with a 20 well comb. High concentration agarose gels should be poured rapidly as the gel solidifies quickly. The gel tray was placed on a wide mini-sub cell GT horizontal electrophoresis system (Bio-Rad, 1704468) and the electrophoresis chamber filled with 1X TAE buffer till about 1 cm above the gel.

10 µl of each PCR sample was loaded into each well and electrophoresis was performed for 1 hour and 10 minutes at 6.7 volts/cm (based on distance between the electrodes). Our power supply (1000/500 power supply, Bio-Rad) was set to 100 V. 4% agarose gels were run at room temperature (25 °C) while 6% gels were run at 4 °C. The percent of agarose used (4–6%) and time of electrophoresis (30 min to 2 h) may need to be adapted to the size of amplicon and electrophoresis apparatus used. A DNA size marker (Gene ruler 1 kB plus, Thermo Scientific, SM1331) was used. No separate dye or loading buffer is needed since the GoTaq green master-mix is a ready-to-use solution containing two dyes. Gel images were acquired using a regular gel-documentation system (Syngene, InGenius3).

### Cloning and sequencing of PCR products

To identify the exact nature of CRISPR-induced mutations, PCR products amplified from DNA of F0 pups were sent for sequencing and mutants showed mixed sequencing peaks (Supplementary Table 1). For confirming the CRISPR induced 7-bp deletion in *Adgrb3*, PCR products of DNA from F1 pups were gel extracted and sub-cloned into pGEMT vectors (Promega, A3600) by TA cloning. Following bacterial transformation, an appropriate number of colonies (usually 8) per F1 mouse were picked and grown overnight. Plasmid DNA was extracted (GeneJET Plasmid Miniprep Kit, Thermo Scientific, K0503) and analyzed by Sanger sequencing (outsourced to Macrogen USA).

### Ethical conduct of research

All mice experiments and procedures were reviewed and approved by the Emory University Institutional Animal Care and Use Committee.

## Results

### Application 1: Initial screening of F0 pups generated from CRISPR-Cas9 microinjected zygotes

We analyzed genomic DNA samples from 47 founder pups derived from CRISPR-Cas9 injected single cell zygotes to detect those carrying genetic alterations in targeted *Adgrb3* locus. PCR products of the target region were directly sequenced (Supplementary Table 1), and DNA of representative wild-type (WT) and all 10 different mutants identified was size-separated by electrophoresis on 4% agarose gels (Fig. 1 and Supplementary Figs S1 and S2). After 1 hr. of electrophoresis amplicons from WT or homozygous mutant mice yielded single bands, which could not be differentiated in size. PCR products of heterozygous mice yielded two distinct bands: a lower one representing homoduplexes (WT/WT or M/M) and an upper one generated by heteroduplexes (WT/M). Interestingly, in two samples (#37 and #39) we could see three bands. F0 mice may harbor biallelic or complicated mosaic mutations, and each type of indel mutation generates a specific heteroduplex migration pattern. The two heteroduplex upper bands in these pups likely originated from the generation of a third mutated allele caused by remnant CRISPR-Cas9 activity beyond the single cell zygote stage. Such resulting mosaic mice may still be of interest if they retain the desired targeted allele in their germline. The complex sequencing peaks of PCR products of samples 37 and 39 (Supplementary Fig. S2) could not be accurately deciphered by the Inference of CRISPR edits software (ice.synthego.com), but their manual alignment with those of WT samples suggested that mouse #37 has a minor allele with an 8-bp deletion along with two WT alleles, while mouse #39 harbors several mutations in two alleles and bears a major third allele with a C/T transition mutation (Supplementary Table 1). These findings are indicative of mosaicism in these F0 pups.

In our CRISPR microinjection experiment the success rate of genetically altered mice was ~23% (11 of 47), so this rapid screen obviated the need for sequencing by 77%. This approach will miss homozygous mutant founders, which are infrequent (our screen had a single one, #15); however, they can be detected by including the second step of our method (see below) in the initial screening. We were also not able to detect a pup (#34) with a 1-bp. insertion.

### Application 2: Genotype offspring of transgenic mice carrying a 7-bp deletion in the *Adgrb3* gene

Next, we examined whether our method could be used for routine genotyping of CRISPR transgenic mice colonies. To test this, we genotyped 7 offspring obtained from a cross of heterozygous mice *(Adgrb3*^+/−^*x Adgrb3*^+/−^) that carry a WT allele and a null allele with a 7-bp deletion.

#### Step 1: Detection of two bands identifies heterozygous mutant mice

**Figure 2:**
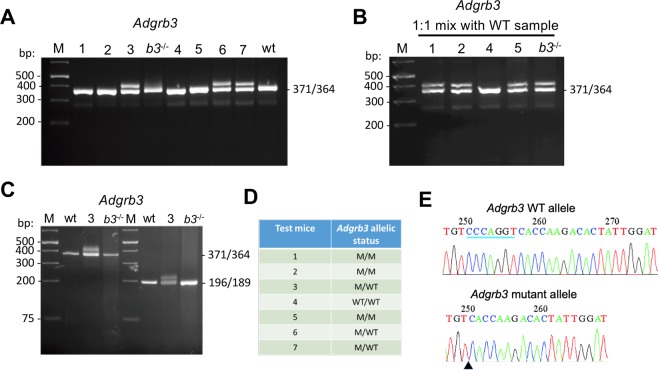
Heteroduplex-based genotyping of 7 mice pups, which are the offspring from a cross between two heterozygous (*Adgrb3*^+/−^*)* mice both of which carry a null allele with a 7-bp deletion in exon 10 of the *Adgrb3* gene. (**A**) STEP1: PCR products were resolved by 4% agarose gel electrophoresis for 1 hour. Amplicons of homozygous WT (371-bp) or mutant (364-bp) mice cannot be distinguished and migrate as a single band. Amplicons of heterozygous mice (#3, 6, 7) generate a mix of 371 and 364-bp products, which hybridize randomly and form homo- and hetero-duplexes during the last cycle of the PCR reaction. The heteroduplexes migrate more slowly in the 4% agarose gel matrix and generate an extra band of apparent size of ~400-bp. PCR products of *Adgrb3* wild-type (wt) and homozygous mutant mice (*b3*^−/−^) previously genotyped by PCR product cloning and sequencing were used as controls. M: DNA size markers. (**B**) STEP2: PCR products of test mice and a control WT mouse were mixed, then denatured/renatured by one extra round of PCR and resolved again by 4% agarose gel electrophoresis. Results show that mice #1, 2 and 5 are homozygous (2 bands), while #4 is wild-type (1 band). (**C**) PCR products of different amplicon sizes (371-bp and 196-bp) and spanning the 7-bp CRISPR deletion region when resolved on a 4% agarose gel. The gel image shows that Heterozygous mutant (#3) yields two bands while control wild-type (wt) and *Adgrb3* homozygous mutant (*b3*^−/−^) produce single bands. (**D**) Independent genotyping by qPCR-based assay (outsourced to Transnetyx.com) confirms allelic status in 7 test mice. Fluorescent signal from *Adgrb3* WT and mutant probes is measured for each allele. Probe 1 (ACCTGGGACATTCC) is specific to the WT allele and binds to 6 of 7 nucleotides that are deleted in the mutant allele. Probe 2 (TGTCTTGGTGACATTC) exclusively binds to the mutant allele. (**E**) Representative chromatograms of PCR product from WT and mutant alleles (with expected 7-bp deletion) cloned into pGEMT vector. The 7-bp. deletion in the *Adgrb3* target region is underlined in the WT allele and its loss is shown by a black triangle in the sequence of the mutant allele.

PCR products of tail snips of the 7 pups were resolved on a 4% agarose gel (Fig. 2A), and double bands were obtained for 3/7 indicating they are heterozygous. The remaining 4 yielded single bands and needed further characterization since they could be due to homoduplexes from WT (371-bp) or 7-bp. deleted alleles (364-bp). To identify them, a second step was performed.

#### Step 2: Hybridization to known WT sample discriminates homozygous WT from homozygous mutants

In the second step, 5 µl of PCR products from the samples yielding single bands in step 1 were mixed individually with an equal volume (5 µl) of PCR product of a known WT sample and subjected to denaturation/renaturation by an additional cycle of PCR. PCR products were then resolved in a second 4% agarose gel. The samples containing mutant alleles formed heteroduplexes with the WT DNA and could be identified as they generated two distinct bands. Mixed WT samples produced homoduplexes and their PCR products migrated as single bands. This analysis showed 3/4 remaining test mice (#1, 2, 5) yielded two bands (Fig. 2B), indicating that they are homozygous mutants, while the last mouse (#4) yielded a single band and is therefore homozygous WT.

To test the effect of amplicon size, we designed primers yielding 196-bp and 100-bp fragments, and observed they were equally efficient in detecting heteroduplexes (Fig. 2C and Fig. 4C).

The genotypes of all 7 test mice were independently confirmed by qPCR based assays (outsourced to Transnetyx) and results are shown in Fig. 2D. We further purified the PCR products with Qiaquick PCR Purification Kit (Qiagen, 28106) and performed TA cloning followed by sequencing of the clones, which confirmed the accuracy of our genotyping method (Fig. 2E).

### Application 3: Genotype offspring of transgenic mice carrying a 3-bp insertion in the *Scn8a* gene

**Figure 3:**
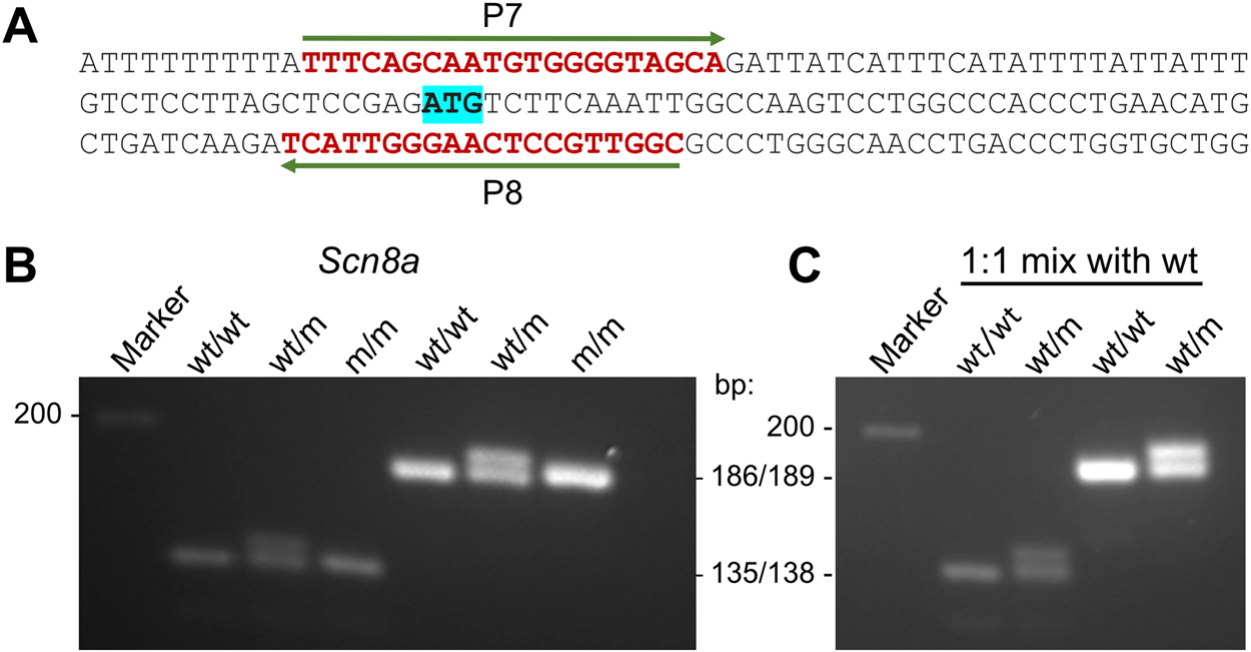
Detection of CRISPR-Cas9 induced 3-bp insertion in the *Scn8a* gene of mice with known genotype. (**A**) The 3 nucleotides insert (ATG) generated by CRISPR-Cas9 in exon 15 of the mouse *Scn8a* gene is highlighted in blue. Regions used to design primers (P7 and P8) used for PCR amplification are indicated (see primer sequences in Table 1). (**B**) STEP1: PCR products of different amplicon sizes (135-bp and 186-bp) were generated by two different sets of primers spanning the CRISPR 3-bp insertion site were resolved on 4% agarose gel electrophoresis. Regardless of primer set used, the heterozygous mutant mouse sample yielded two distinct bands. (**C**) STEP2: PCR products of samples producing a single band in step 1 were mixed with PCR products of control WT sample (1:1 by volume) and denatured/renatured by one additional cycle of PCR and resolved again by 4% agarose gel electrophoresis. The homozygous mutant now yields two distinct bands, while the wild-type sample produces a single band.

**Figure 4:**
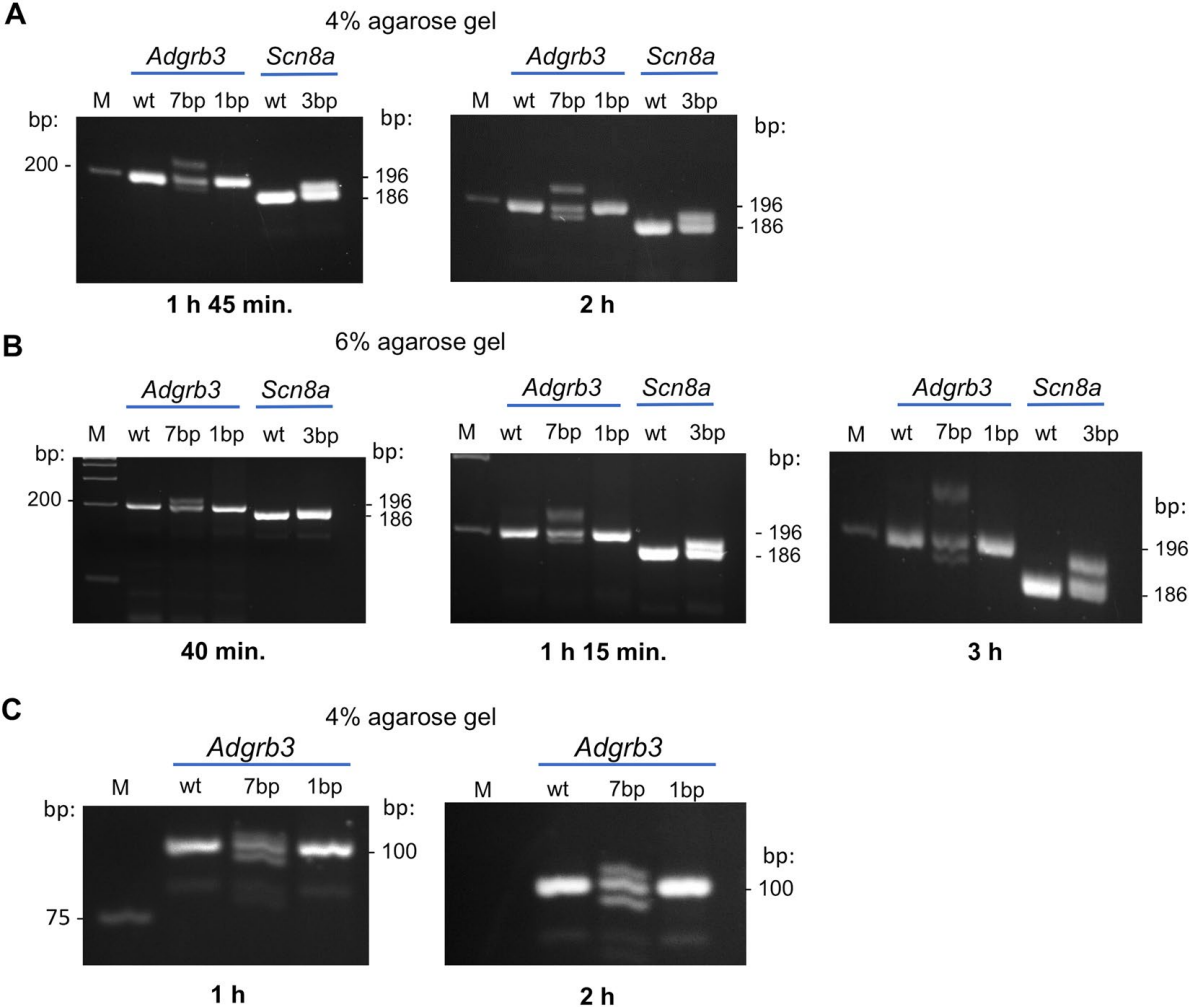
Effect of time of migration and concentration of agarose on heteroduplex band resolution. Time course of electrophoresis of PCR products of samples with 7-bp deletion (#3) and 1-bp insertion (#34) in the *Adgrb3* gene (196-bp amplicon), and 3-bp insertion in the *Scn8a* gene (186-bp amplicon) on 4% (**A**) and 6% (**B**) agarose gels. Note: When PCR products of the 7-bp and 3-bp heterozygous mutants were run longer (3 h) on 6% agarose gels 3 bands were obtained, showing the wt/wt homoduplexes could be separated from the mut/mut homoduplexes. (**C**). Gel image showing the effect of migration time and shorter amplicon size (100bp) on resolution of 7-bp and 1-bp alterations in *Adgrb3* in 4% agarose gels. M: DNA size marker.

To test our method on a smaller alteration and a different gene, we analyzed genomic DNA from a transgenic mouse line with a CRISPR-Cas9 generated 3-bp insertion in exon 15 of the *Scn8a* gene. Two sets of primers yielding two different amplicon sizes (135 and 186-bp) were used. PCR products from heterozygous mice generated two bands, while those of homozygous WT and mutant mice yielded single bands (Fig. 3A). The second step revealed a double band only in homozygous mutant samples while the WT continued to yield a single band as expected (Fig. 3B). Hetero and homo-duplexes were easily resolved after 1 hour of electrophoresis in 4% agarose gels and use of 6% agarose gels could accelerate the detection even further (~30 min.). Longer gel migration (2 h in 4% and 1 h 15 min in 6% agarose gels) could even distinguish WT from mutant homoduplexes, with indels as small as 3-bp (Fig. 4).

Together, our data demonstrate that this high concentration agarose electrophoresis based genotyping approach can identify both insertion and deletion CRISPR mutations in different genes in mice.

## Discussion

Discriminating the size of PCR products from WT versus mutant alleles with small indels in CRISPR targeted genes is beyond the size resolution capacity of standard 1% agarose gel electrophoresis. Depending on the nature of indels, genotyping CRISPR-Cas9 generated transgenic animals requires sophisticated techniques such as Sanger sequencing of PCR products, T7 endonuclease-I (T7E1) cleavage assay^4,14^, PAGE based detection of heteroduplexes^5,14^, or qPCR based assays^6^. As an alternative, we have developed a simple two-step method for genotyping CRISPR-Cas9 modified alleles by analyzing hetero- and homoduplexed DNA hybrids in high concentration agarose gels. Indels in one or both alleles can be rapidly detected using PCR amplification of the CRISPR target region followed by 4–6% agarose gel electrophoresis of the PCR products. A widely used assay for indel detection is the T7E1 cleavage assay, which is based on recognition and cleavage by T7 endonuclease 1 (T7E1) of distorted (kinked structure) heteroduplex dsDNA^15^. This method requires pre-testing of PCR products on a 1.5% agarose gel, separate hybridization of PCR products, and enzymatic reaction. Our method does not require such steps, saving time and avoiding false negative results due to incomplete digestion of mismatched DNA fragments.

Our assay involves direct gel visualization of hetero-/homo-duplexes formed after PCR amplification of CRISPR-Cas9 target regions following high concentration agarose gel electrophoresis. Results are obtained rapidly (~5 hr. turnaround time, including sample and gel preparation, completion of PCR and resolving PCR products on agarose gels) and the method can be performed in any standard laboratory worldwide using a regular thermal cycler and agarose gel electrophoresis instrumentation. We showed the method is applicable to different CRISPR-Cas9 targeted genes and detection is faster than PAGE based methods, which require 15% polyacrylamide gels^5^, are time-consuming to prepare, require at least 2 hours of electrophoresis, and use neurotoxic and carcinogenic chemicals. We tested this new method as an initial screening tool to detect F0 pups with different types of indel mutations generated from CRISPR-Cas9 microinjected single cell embryos and were able to detect all mice with genetic alterations except one with a 1-bp. insertion, showing a limitation. The analysis also revealed mosaic indel mutations since heteroduplexes with differential migration patterns were observed during initial screening of single F0 pups. Such mosaic animals are likely generated from remnant CRISPR activity after the single cell embryo stage, generating additional mutated alleles at the 2–4 cell stage.

These findings suggest this method is applicable to detect a wide array of small indel mutations of >2-bp. in alleles from CRISPR-Cas9 modified animals. Whether it can detect 2 bp. indels remains to be investigated. A recent study interrogating the molecular effects of CRISPR-Cas9 mediated deletions in targeted sites of 632 founder mice and 54 already established CRISPR mice lines revealed that the median deletion size using single sgRNAs is 9-bp.^16^. For projects aiming to create 1–2 bp indels a PAGE based method or direct Sanger sequencing of PCR products is still indicated.

The method will be particularly useful for routine genotyping of offspring of already established transgenic mice colonies as we showed for mice harboring *Adgrb3* (7-bp. deletion) and *Scn8a* (3-bp. insertion) mutant alleles. Hetero and homo-duplexes were easily resolved within 0.5-1 hour of electrophoresis in 4-6% agarose gels and longer gel migration even allowed for separation of WT from mutant homoduplexes. To date, we have genotyped over 90 mice and found highly reproducible results.

In summary, we present a simple, cost effective, and time saving genotyping method that can be implemented with standard molecular biology equipment and applied to screen for indels in founder pups of CRISPR-Cas9 generated transgenic animals and also to genotype offspring in established transgenic animal colonies. While we did not yet try to genotype CRISPR-Cas9 modified cell lines, this method will likely be applicable with the possible caveat that aneuploidy may complicate the analysis.

## Supplementary information

Supplementary Dataset 1

## Data Availability

The authors confirm that all data supporting this method are available within the article and its supplementary materials and also with the authors and are available upon request.
