# Supplementary material for: A simple genotyping method to detect small CRISPR-Cas9 induced indels by agarose gel electrophoresis
Source: Sci Rep. 2019 Mar 14;9:4437. doi: 10.1038/s41598-019-39950-4 (PMC6418129; doi:10.1038/s41598-019-39950-4)
Supplement: Supplementary file 1 — Supplementary Dataset 1 [file 41598_2019_39950_MOESM1_ESM.pdf]

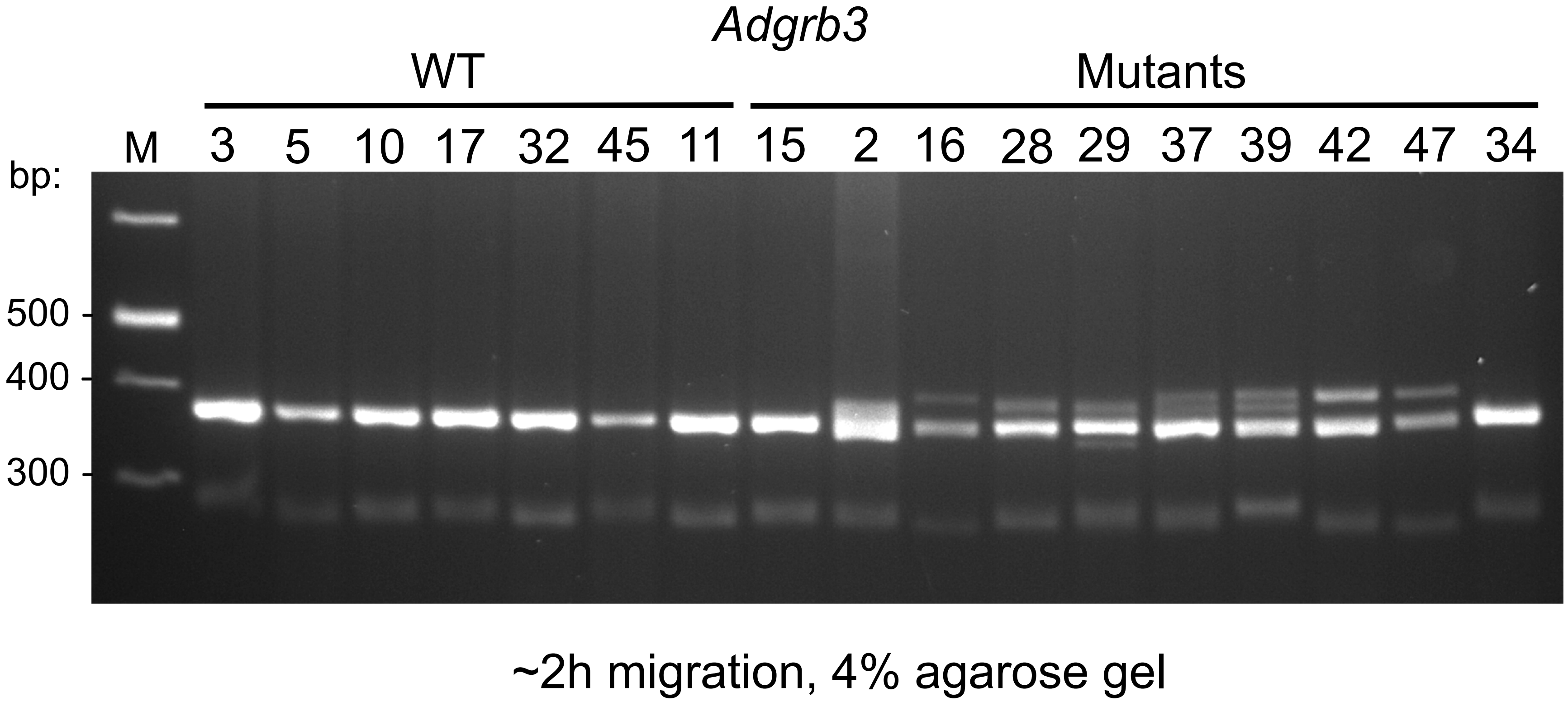

Figure S1. Screening of F0 pups generated from *Adgrb3* targeted CRISPR microinjected zygotes to detect potential heterozygous founder animals. Representative gel image shows screening of 17 F0 pups (7 wild-type and 10 mutants). CRISPR target *Adgrb3* locus was PCR amplified from genomic DNA of pups using primers P1 and P2 and PCR products were run on a 4% agarose gel for 2 h. Sample 15 is a homozygous mutant with a 7-bp deletion (see Supplementary Table 1), so migrates as a single band.

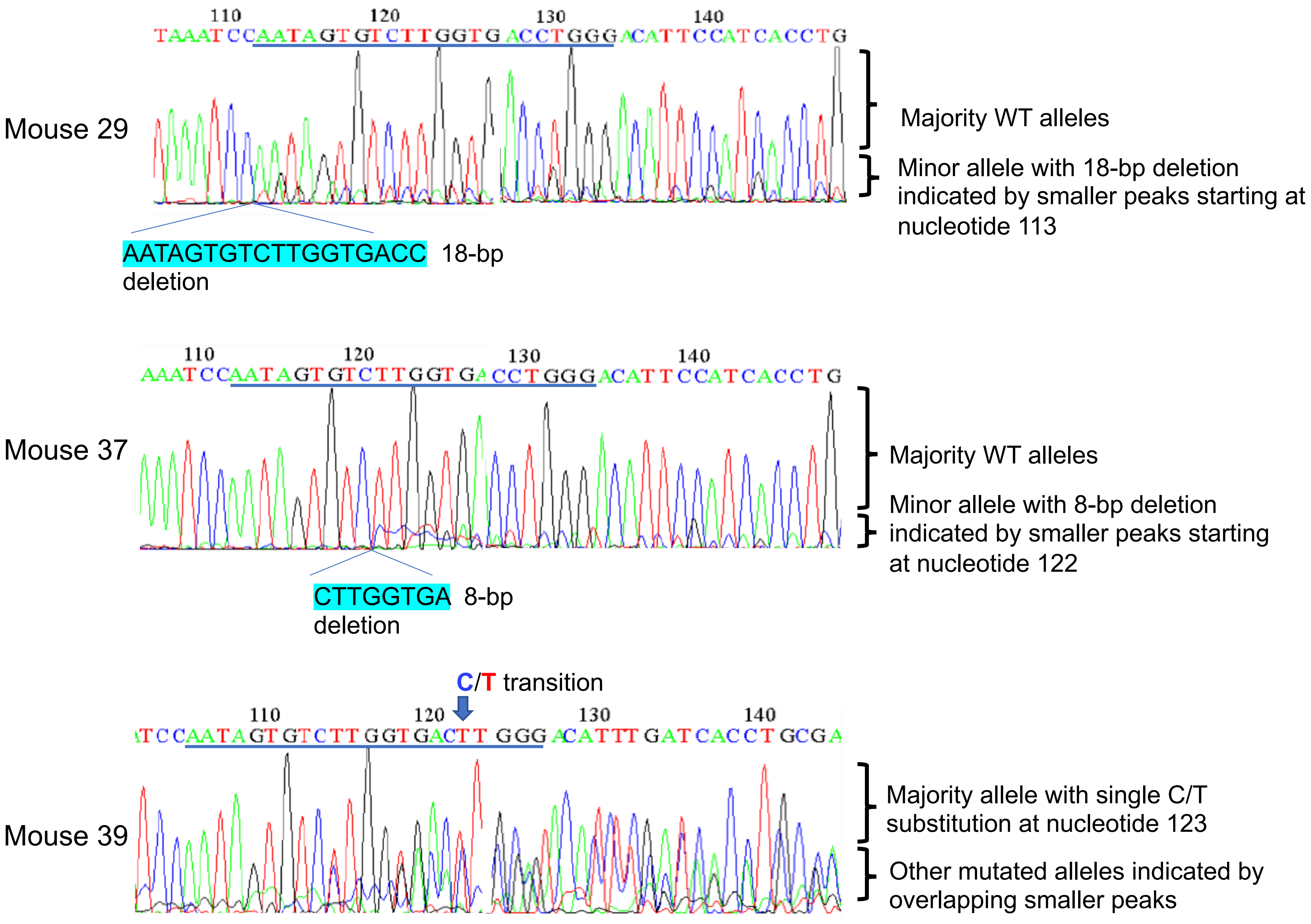

Figure S2. Sequencing of PCR amplicons of founder pups (# 29,37,39). The CRISPR target region in exon 10 of the *Adgrb3* gene is underlined. CRISPR-generated deletions are highlighted in blue and their relative location in the amplified sequence are indicated.

Supplementary Table 1

Mouse #	Allele 1	Allele 2	Allele 3	Zygosity	Altered sequence
1	WT	WT	N/A	Homozygous	N/A
2	WT	9 bp deletion	N/A	Heterozygous	TGGTGACCT
3	WT	WT	N/A	Homozygous	N/A
4	WT	WT	N/A	Homozygous	N/A
5	WT	WT	N/A	Homozygous	N/A
6	WT	WT	N/A	Homozygous	N/A
7	WT	WT	N/A	Homozygous	N/A
8	WT	WT	N/A	Homozygous	N/A
9	WT	WT	N/A	Homozygous	N/A
10	WT	WT	N/A	Homozygous	N/A
11	WT	WT	N/A	Homozygous	N/A
12	WT	WT	N/A	Homozygous	N/A
13	WT	WT	N/A	Homozygous	N/A
14	WT	WT	N/A	Homozygous	N/A
15	7bp deletion	7bp deletion	N/A	Homozygous	ACCTGGG
16	WT	7bp deletion	N/A	Heterozygous	ACCTGGG
17	WT	WT	N/A	Homozygous	N/A
18	WT	WT	N/A	Homozygous	N/A
19	WT	WT	N/A	Homozygous	N/A
20	WT	WT	N/A	Homozygous	N/A
21	WT	7bp deletion	N/A	Heterozygous	ACCTGGG
22	WT	WT	N/A	Homozygous	N/A
23	WT	WT	N/A	Homozygous	N/A
24	WT	WT	N/A	Homozygous	N/A
25	WT	WT	N/A	Homozygous	N/A
26	WT	WT	N/A	Homozygous	N/A
27	WT	WT	N/A	Homozygous	N/A
28	WT	8bp deletion	N/A	Heterozygous	CTTGGTGA
29	WT	WT	18bp deletion	Mosaic	AATAGTGTCTTGGTGACC
30	WT	7bp deletion	N/A	Heterozygous	ACCTGGG
31	WT	WT	N/A	Homozygous	N/A
32	WT	WT	N/A	Homozygous	N/A
33	WT	WT	N/A	Homozygous	N/A
34	WT	1bp insertion	N/A	Heterozygous	C
35	WT	WT	N/A	Homozygous	N/A
36	WT	WT	N/A	Homozygous	N/A
37	WT	WT	8bp deletion	Mosaic	CTTGGTGA
38	WT	WT	N/A	Homozygous	N/A
39	C/T transition	Mutation (unclear)	Mutation (unclear)	Mosaic	C>T transition, other mutations unclear.
40	WT	WT	N/A	Homozygous	N/A
41	WT	WT	N/A	Homozygous	N/A
42	WT	9bp deletion	N/A	Heterozygous	TGGTGACCT
43	WT	WT	N/A	Homozygous	N/A
44	WT	WT	N/A	Homozygous	N/A
45	WT	WT	N/A	Homozygous	N/A
46	WT	WT	N/A	Homozygous	N/A
47	WT	7bp insertion	N/A	Heterozygous	ATTCCAT

Supplementary Table 1: Sequencing results of PCR products of the entire cohort of 47 F0 pups born from pseudo-pregnant females implanted with single cell zygotes microinjected with an *Adgrb3*-targeted CRISPR/Cas9. Altered sequences in each mutant are shown in the last column. Three mice (#29, 37, 39) showed evidence for more than 2 alleles, suggesting mosaicism (see Figure S2). N/A: not applicable.
